# Case of a Very Old and Irreducible Entero-Epiplocele, with Adhesion to Its Sac, That Was Cured in Three Weeks by Confinement to Bed in the Horizontal Position

**Published:** 1820-10

**Authors:** R. W. Bampfield

**Affiliations:** Surgeon.


FOR THE LONDON MEDICAL AND PHYSICAL JOURNAL.

### *Case of a very old and irreducible Entero-Epiplocele, with Adhesion to its Sac, that was cured in three Weeks by Confinement to Bed in the Horizontal Position.* By R. W. Bampfield, Esq. Surgeon.

IT may have occurred to many surgeons, to observe the gradual recession of an old and irreducible hernia within the abdomen, during the long confinement of a patient (so affected) to bed, from other causes;* but the records of surgeons do not furnish many instances of patients submitting, by their advice, to a long-continued horizontal posture for the purpose of this gradual reduction. M. Arnaud† reduced several old herniæ in two or three weeks by a severe treatment, which is confirmed by the testimony of Le Dran, (p. 84, Gataker's translation.) Mr. Hey‡ mentions several, which "had not contracted any adhesion to the hernial sac," and were probably recent cases. They were, in general, cured in five or six weeks, and one was so in one week. The following case having terminated successfully in three weeks, may not be unworthy of record or the practice of imitation.

Mr. F—, æt. 42, was affected with a double scrotal hernia, to support which he had only employed the common bag-truss; but, being alarmed at the fatal issue of a case similarly situated with his own, he determined to consult some experienced surgeon. The right rupture contained intestine, was reducible, and of one year's duration. The left was irreducible, was of the size of a large fist; and this irreducible part consisted of omentum adhering to its sac, the prolapsed intestine on this side having been returned by the first attempt: it had been of such long duration, that the period of its protrusion had escaped the memory; for two years, however, it had been troublesome, painful, and, during the late summer-heat, had increased in size, apparently from a recent descent of intestine.

---

* Hildanus, Cent. 5, Obs. 54; Pott, p. 57.

† *On Hernia*, p. 292.

‡ P. 222, 3d edit.

On Saturday, the 5th of August, he held a consultation at Mr. Brodie's house, with his medical friend Mr. Prower, and was advised to wear a double truss, the left pad of which was to be allowed to press on the unreduced omentum.* In the evening, being desired to visit the patient, and having examined him in bed on the following morning, I advised him to continue in bed, and await the result of a consultation with Mr. Brodie on Monday. This consultation turned entirely on the chances of the omentum and its sac retiring within the abdomen during a long-continued horizontal posture; and we agreed to advise him to try it for two months, to which he readily consented. Mr. B. attended in consultation once a-week on the subject of the hernia; while I visited the patient every day, and at least four times a-week perseveringly employed the taxis, as long as he could patiently endure the degree of pain it occasioned; and he was enjoined to support the omentum, and employ as much pressure with his hands, as often as he could, in the direction of the passage from the outer to the inner ring, which he performed with intelligence and industry. To obviate constipation, and to lessen the contents of the abdomen, a draught of ol. ricini was given twice a-week. In a fortnight, the rupture was more than half reduced; and Mr. W. Sheldrake was recommended by Mr. Brodie to fit a bag-truss so as to sustain the omentum as high in the scrotum as possible; but it was of no evident advantage.

On the 27th of August, so much of the omentum was returned by the taxis, that I could press the ball of my thumb into the outer ring, so as to confine the omentum and its sac between it and the inner ring; but the patient endured so much pain about the inner ring, that I was obliged to desist from attempting its entire reduction: the patient, however, was directed to retain it in that reduced position as much as he could. On the 28th, I could easily return the omentum and sac within the outer ring; but the attempt to press it forcibly and entirely into the abdomen occasioned the same pain as yesterday: the patient was requested to retain it in that position, and, after about fifteen minutes' perseverance, it passed into the abdomen with considerable pain, which was most probably produced by its dragging-in the spermatic chord with it, to which it had adhered, and by its raising the testis as high as the ring; from which it gradually descended in a few days. The patient was not restricted in diet, and his general health did not suffer from his three weeks' confinement.

* Trusses with hollow pads have succeeded in omental bubonocele; but, says Le Dran, "this method will be of no service in any hernia where the epiploon has fallen into the scrotum," p. 88: a statement that is confirmed by all succeeding writers.

As reason and experience allow us to assume, that parts which have often passed through an elastic tube can be made to repass it, so they permit us to infer that many old herniæ, termed irreducible, admit of a fortunate reduction. The parts through which a rupture makes its transit, are considerably, though slowly, dilatable, or they never could have stretched to such dimensions as we occasionally find them. There does not appear to be any necessity for the frequent bleedings, the active purgatives, and very low diet of Arnaud; in a word, for the system of making skeletons of living bodies: for, if the recumbent posture be submitted to; if frequent pressure with the hands be used;* if the bowels be kept free from constipation; and the taxis often employed with perseverance, we see no cause why success should not be attained: an event that may be soon ascertained; for, if a portion can be returned, we may rest assured the continuance of the same means will effect a complete reduction.

*37, Bedford-street, Covent Garden; Sept. 6th,* 1820.

* This is thought much preferable to Mr. A. Cooper's laced suspensory.

